# *Keylimepie peckorum* gen. n. and sp. n., (Hymenoptera, Braconidae) from southern Florida, U.S., the first known brachypterous member of the subfamily Microgastrinae

**DOI:** 10.3897/zookeys.584.8319

**Published:** 2016-04-26

**Authors:** Jose Fernandez-Triana, Caroline Boudreault

**Affiliations:** 1 Canadian National Collection of Insects, 960 Carling Ave., Ottawa, ON K1A 0C6 Canada

**Keywords:** Microgastrinae, new genus, North America, southern Florida, *Keylimepie*, brachyptery, conservation

## Abstract

*Keylimepie peckorum* Fernandez-Triana, **gen. n.** and **sp. n.**, are described from southern Florida, U.S. Females have the shortest wings (0.6–0.7 × body length) of any known microgastrine wasp. The genus can also be recognized on features of the head, propodeum and first three metasomal tergites. All specimens were collected in hammock forests of the Florida Keys and Everglades National Park, but their host caterpillar is unknown. Because its morphology is unique and it is the first new microgastrine genus discovered in North America since 1985, the potential for future conservation of the species is discussed.

## Introduction

Microgastrinae (Hymenoptera, Braconidae) is a hyperdiverse and challenging group of parasitoid wasps, with almost 2,700 described species (Yu et al. 2012, Quicke 2015, Fernandez-Triana and Ward 2016). This subfamily is most diverse in tropical areas, where thousands of species and several new genera remain undescribed. The Nearctic fauna is better studied, although it is far from being completely inventoried, with numerous new species awaiting discovery. The last two new genera of Microgastrinae described from North America were *Pelicope* (Mason, 1981) and *Lathrapanteles* (Williams, 1985), more than 30 years ago. The finding of a new genus in this region is unexpected. As part of ongoing studies on the diversity of Microgastrinae in Florida, a species with unusually short wings was discovered among samples from the Florida Keys and Everglades National Park. They represent the first known brachypterous species of Microgastrinae, and the first new genus found in North America since 1985.

## Methods

All 60 specimens studied for this paper were found among unsorted Microgastrinae in the Canadian National Collection of Insects (CNC).

Morphological terms and measurements of structures are mostly as in Mason (1981), Huber and Sharkey (1993), Whitfield (1997), Karlsson and Ronquist (2012), and Fernández-Triana et al. (2014). Mediotergites 1, 2, and so on, are abbreviated as T1, T2, etc.; ocular ocellar line as OOL and posterior ocellar line as POL.

Photos were taken using a Canon EOS 60D with MPE-65 lenses (aperture: 4.0, ISO: 100, CR2 format images) and a 600EX-RT Speedlight (manual) flash. Multiple images through the focal plane were taken of a structure, and these were combined to produce a single in-focus image with Zerene Stacker (http://zerenesystems.com/cms/stacker). Plates for the illustrations were prepared using Adobe Photoshop CS4.

A map with the distribution of all species was generated using SimpleMappr (Shorthouse 2010).

## Results

### Keylimepie

Taxon classificationAnimaliaHymenopteraBraconidae

Fernandez-Triana gen. n.

http://zoobank.org/B1AC9A3C-1368-42AF-9A34-9DDC72867C80

#### Type species.

*Keylimepie peckorum* Fernandez-Triana new species, by present designation.

#### Diagnosis.

Female. Fore wings (0.6–0.7 × body length), shorter than any other species of Microgastrinae (where fore wings are 0.9–1.2 × body length); malar distance more than 2.0 × mandibular width; eye and ocelli small. Both sexes. Propodeum sculpture with a complex pattern that includes partial transverse and longitudinal carinae and a posteriorly defined areola; T1 without median sulcus; T1–T3 with strong longitudinal sculpture.

#### Description.

Female. Head heavily punctured, in lateral view strongly projecting forward below the antennal sockets. Malar distance more than 2.0 × mandibular width. Anterior tentorial pits very large compared to clypeus width. Eyes and ocelli small. Pronotum with one broad, transverse pronotal sulcus. Propleuron flange sharply defined by carina. Notauli faint, almost invisible. Propodeum with complex sculpture pattern, including partial median longitudinal carina (sometimes obscured by other sculpture), traces of transverse carina, and partial areola with only the posterior carina defined. Fore wing short and narrow, brachypterous (fore wing length 0.6-0.7 × body length, and 1.1–1.3 × metasoma length), with small to almost obliterated quadrangular areolet. Metacoxa of moderate size, not surpassing posterior margin of T2. Inner and outer spurs of metatibia subequal and less than half length of metatarsal segment 1. T1 relatively short, more or less parallel-sided in anterior 0.5, then narrowing towards posterior margin, without median sulcus, with anterior 0.5 rather depressed and concave, and posterior 0.5 with strong transversal striation. T2 trapezoidal. T2 and T3 with strong longitudinal striations, T4–T8 smooth. Hypopygium small, inflexible and unfolded. Ovipositor sheaths mostly smooth, with a few, short setae posteriorly. Ovipositor very short, 0.2 × as long as metatibia. Male as in female but not brachypterous, antenna uniformly brown and body color much darker.

#### Distribution.

Southern Florida: four Florida Keys and a single locality in Everglades National Park (Fig. 18).

#### Hosts and habitat.

Hosts are unknown. All specimens were collected in hammock forests from February to November.

#### Putative autapomorphies.

Propodeum carination pattern, shape and sculpture of T1 (without median sulcus), sculpture of T2 and T3, reduced eyes, long malar space, and, in females, short fore wing are treated tentatively as apomorphies.

#### Etymology.

Named after the Key Lime Pie, a typical dessert originated in the Florida Keys –the same area where the new genus was collected. The name not only honors a significant component of the culinary culture in southern Florida, but is also intended to bring attention to and promote conservation efforts for the habitats where the wasps occur. The gender of the genus name is neuter.

#### Comments.

The relationships of *Keylimepie* with other genera of Microgastrinae are hard to assess at present, especially because there are no molecular data available, and nothing is known about potential hosts. Several morphological characters, e.g., head shape and sculpture, mesosoma sculpture, shape and sculpture of T2, and ovipositor, suggest it is related to some species-groups of *Diolcogaster*, part of the Cotesini tribe (sensu Mason 1981). That may be the best placement for the time being and it is the one we favour here. However, several other characters are different from the traditional Cotesini, e.g., the relatively short metacoxa and short metatibial spurs, shape of T1 (and lack of median sulcus), and carination pattern of propodeum. Regardless of affinities to other Microgastrinae genera, *Keylimepie* is a distinctive genus.

The reduced wings in females, relatively small eyes and long malar space present interesting evolutionary questions. More study of additional species of Microgastrinae worldwide would be needed before it can be established if those characters truly are autapomorphies of *Keylimepie* or just an adaptation to local conditions (see Discussion below).

### Keylimepie peckorum

Taxon classificationAnimaliaHymenopteraBraconidae

Fernandez-Triana sp. n.

http://zoobank.org/BFD41F88-9B9F-4088-94D4-200BB7FE9BC1

Figs 1–6 , 7–12 , 14–17

#### Holotype.

Female, CNC. UNITED STATES, Florida, Monroe County, North Key Largo, Sec., 25°17'23"N, 80°18'25"W, 3.IV.1985, Malaise trap, S. & J. Peck (colls). Holotype voucher code: CNC483649.

#### Paratypes.

3 ♀, 56 ♂ (CNC). United States, Florida, Monroe County. All specimens collected by S. & J. Peck in the following localities and dates: Big Pine Key, Watson’s Hammock, 24.716667 -81.304167, 28.viii.1986, voucher code CNC483646; Everglades National Park, Royal Palm Hammock, 25.381667 -80.609722, Malaise trap, 3.iv.1985, coll., voucher codes CNC483647, CNC483648; Fat Deer Key, 24.735344 -81.011853, 18.x.1985-25.ii.1986, voucher codes CNC483464, CNC483465, CNC483468; Malaise trap, 18.xi.1985-25.ii.1986, voucher codes CNC489922, CNC489923, CNC489928, CNC489929, CNC489931–CNC489933, CNC489937, CNC489938, CNC489940, CNC489941, CNC489944, CNC489945, CNC489947; 2.viii-16.xi.1985, voucher codes CNC489955, CNC489959, CNC489962, CNC489965; 24.ii-4.vi.1985, voucher codes CNC483624, CNC483625; 3.iv.1985, voucher codes CNC483627, CNC483630, CNC483634–CNC483636; 4.v-4.viii.1985, voucher codes CNC489874, CNC489876, CNC489877; Malaise trap, iii.1985, voucher codes CNC483626, CNC483628, CNC483629, CNC483631–CNC483633; Fat Deer Key, 24.735344 -81.011853, Hammock, Malaise trap, 24.ii-4.vi.1986, voucher code CNC491265; North Key Largo, Sec., 25.289722 -80.306944, Malaise trap, 3.iv.1985, voucher code CNC483615; N. Key Largo, Sec., 25.289722 -80.306944, Malaise trap, 3.iv.1985, voucher codes CNC483637–CNC483645, CNC483649; North Key Largo, S35, 25.289722 -80.306944, Hammock forest, Malaise trap, 4.v-4.viii.1985, voucher codes CNC483452, CNC483455, CNC483456, CNC483458; Sugar Loaf Key, Kichings, 24.644000 -81.563000, Malaise trap, 3.v-3.viii.1985, voucher codes CNC489899, CNC489907, CNC489913; Vaca Key, Marathon, 24.716936 -81.073308, flight interception trap, 31.viii-15.xii.1986, voucher code CNC483448.

#### Description.

**Female.** **Color.** Body mostly orange yellow, with mediotergites 4–8, laterotergites 4–8, sternites 4–8 and hypopygium darker, mostly brown. Antenna with anterior 6–7 flagellomeres yellow, and posterior 9–10 flagellomeres dark brown. Metatibia and metafemur mostly to completely brown. Anteromesoscutum, propodeum and metapleuron varying from entirely orange-yellow to partially dark brown. Wings mostly infumated, except for small central white band; veins mostly brown, pterostigma with a pale spot anteriorly or mostly brown.

**Head.** Coarsely sculptured, in lateral view strongly projecting forward below antennal sockets. Head in frontal view with maximum width right below the eyes (due to bulging gena). Gena in lateral view wider than eye width. Anterior tentorial pits large, 0.3 × as wide as clypeus width. Malar line long, more than 2.8 × mandibular width. Eye small, its height 0.6 × head height. Ocelli relatively small, diameter of posterior ocelli about half of both OOL and POL. Anatomical line tangent to posterior margin of anterior ocellus, crossing well above the anatomical line tangent to anterior margin of posterior ocelli. Antenna about same length as body.

**Mesosoma.** Pronotum rather narrow, with one broad, transverse pronotal sulcus. Propleuron flange sharply defined by carina. Notauli very faintly marked by slightly coarser sculpture than rest of anteromesoscutum. Scutoscutellar sulcus with seven carinae. Mesoscutellum with lateral face mostly strongly striated, with polished area (mesoscutellum lunula) very narrow, 0.1 × lateral face height. Scutellum with posteromedian band weakly rugose. Mesopleuron sculptured on anterior half, smooth posteriorly. Mesopleural scrobe extending to almost 0.5 of mesopleuron width. Metapleuron mostly smooth anterior to metapleural scrobe but with strong transverse striation posterior to scrobe. Propodeum with complex sculpture pattern, including partial median longitudinal carina (sometimes obscured by other sculpture), traces of transverse carina and partial areola with only the posterior carinae of areola defined.

**Metasoma.** T1 relatively short, its medial length 1.2 × its width at anterior margin. T1 more or less parallel-sided for anterior 0.5, then narrowing towards posterior margin (width at anterior margin 2.0 × width at posterior margin). T1 without median sulcus, with anterior 0.5 rather depressed and concave, and posterior 0.5 with strong transversal striation. T2 trapezoidal, its width at posterior margin 1.5 × its medial length. T2 and T3 with strong longitudinal striation; T4–T8 smooth. Hypopygium small, inflexible and unfolded. Ovipositor sheaths mostly smooth, with few, short setae on posterior 0.2–0.3. Ovipositor short, 0.2 × as long as metatibia.

**Legs.** Metacoxa of moderate size, not surpassing posterior margin of T2; metafemur 5.0 × as long as wide. Metatibia with inner and outer spurs subequal (inner 1.1 × as long as outer) and 0.35–0.40 as long as metatarsal segment 1.

**Wings.** Fore wing relatively short (0.6–0.7 × body length and 1.1–1.3 × metasoma length), with small to almost obliterated, 4-sided areolet, and with vein R1 shorter than pterostigma length and only 2.0–2.5 × as long as the distance between its posterior end and posterior end of vein M.

**Holotype measurements** (some measurements of female paratypes between parentheses). Body length: 2.2 mm (1.9– 2.1, 2.4–2.5 mm). Fore wing length: 1.3 mm (1.2, 1.6–1.8 mm). Metasoma length: 1.1 mm (0.9–1.0, 1.2–1.3 mm). Mandible width: 0.06 mm. Malar line: 0.17 mm. Clypeus length/ width: 0.15 mm/0.045 mm. Tentorial pit width: 0.05 mm. Head maximum width: 0.58 mm. Head height: 0.48 mm. Eye height: 0.27 mm. Eye maximum width (lateral view): 0.15 mm. Gena maximum width (lateral view): 0.20 mm. OOL: 0.09 mm; POL: 0.09 mm; diameter of posterior ocellus: 0.05 mm. Flagellomere 2 lenght/width: 0.20 mm/0.065 mm. Flagellomere 14 length/width: 0.14/0.07. T1 width at anterior margin/width at posterior margin: 0.39 mm/0.19 mm. T2 length/width at posterior margin: 0.34 mm/0.52 mm. Metacoxa length: 0.42 mm. Metafemur length/width: 0.75/0.15. Metatibia length: 0.82 mm. Metatibial spurs length, inner/outer: 0.17 mm/0.15 mm. First segment of metatarsus length: 0.40 mm. Ovipositor length: 0.15 mm.

**Figures 1–6.**
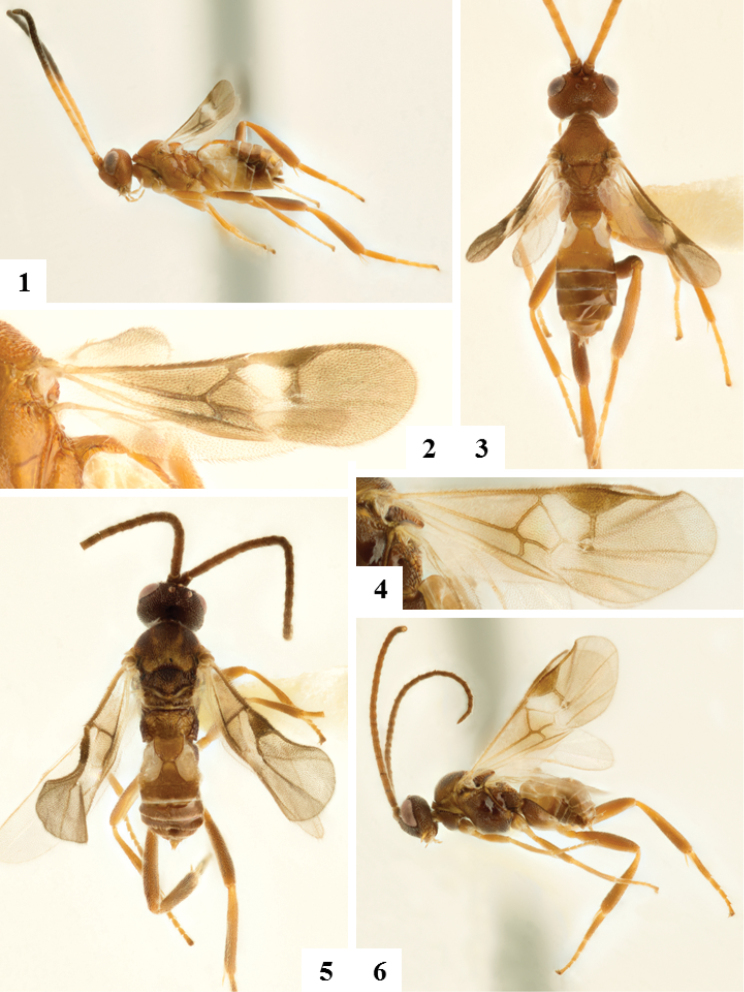
**1–3** Female holotype. **1** Habitus lateral **2** Fore wing **3** Habitus dorsal **4–6** Male paratype **4** Fore wing **5** Habitus dorsal **6** Habitus lateral..

**Figures 7–12.**
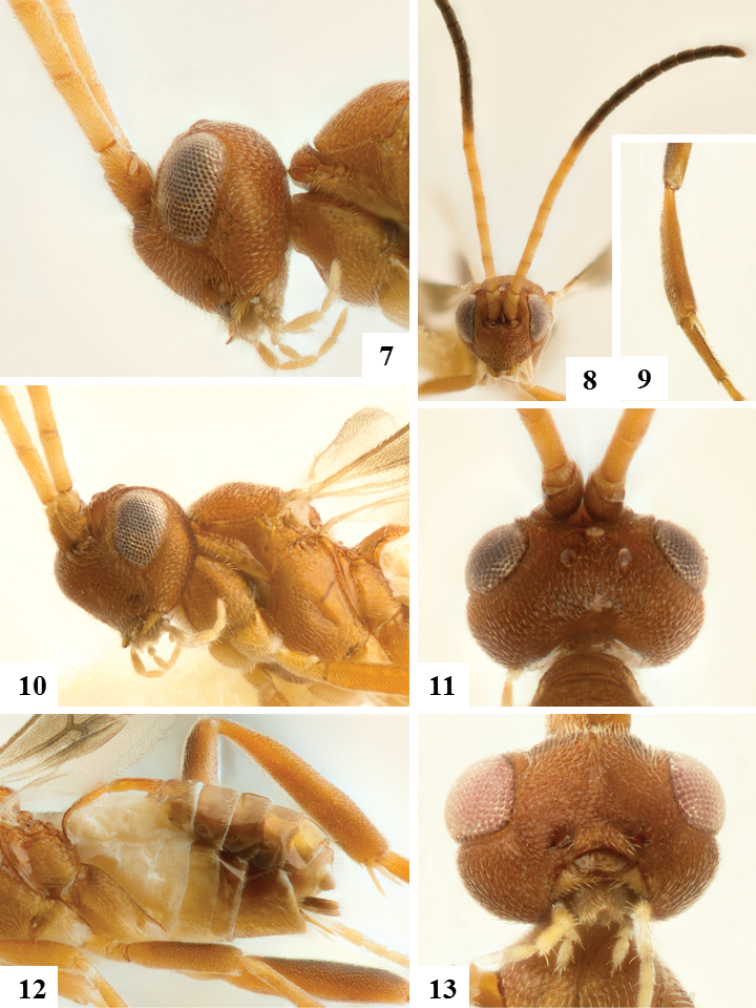
Female holotype. **7** Head, pronotum and propleuron, lateral **8** Head and antenna, frontal **9** Metatibia and first segment of metatarsus **10** Head and mesosoma, dorso-lateral **11** Heard, dorsal **12** Metasoma, lateral **13** Female paratype. Head, ventral.

**Figures 14–17.**
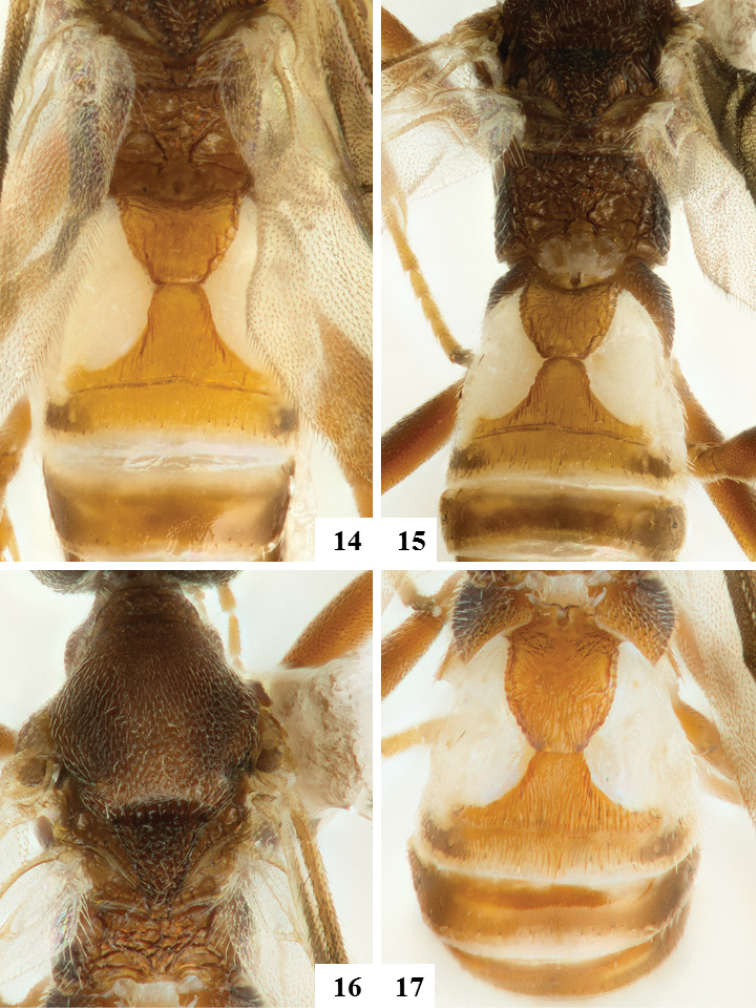
**14–16** Female paratypes. **14** and **15** Propodeum and metasomal tergites 1–4, dorsal **16** Mesosoma, dorsal **17** Female holotype. Metasoma, dorsal.

**Male.** As in female except for uniformly brown antenna, longer fore wing, lighter-coloured wings (hyaline or slightly infumated), and darker body color which in some cases becomes dark brown to black in areas of the head and mesosoma.

#### Distibution.

**Figure 18.**
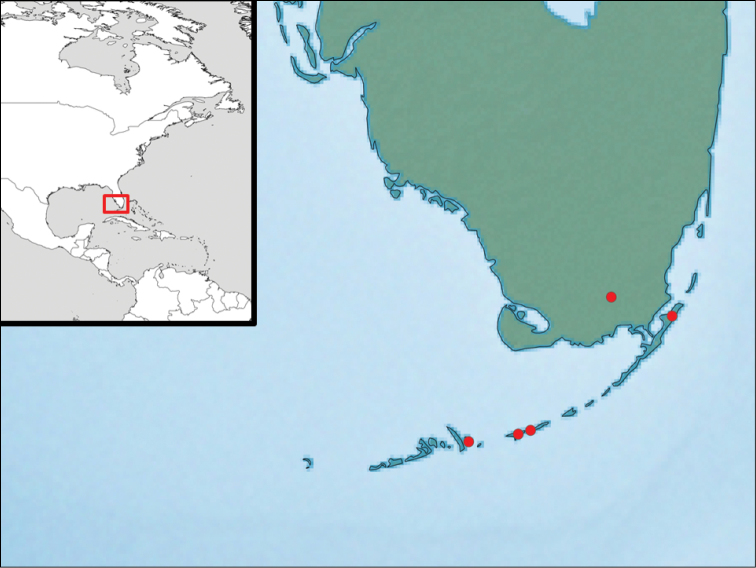
Map showing the distribution of the species.

Southern Florida (Florida Keys and Everglades National Park, Fig. 18).

#### Biology.

Unknown. All specimens were collected in hammock forests from February to November. Based on the labels, the species would seem to be more abundant in April–June and November. However, the collecting device, a Malaise trap, was not emptied regularly (at times running for months), and so the actual flight dates for the species cannot be considered as very precise.

#### Etymology.

Named after Stewart and Jarmila Peck, tireless insect collectors over the last 30+ years and the ones finding the species back in 1985 and1986. Also in recognition of their great knowledge as entomologists and as an appreciation for the important papers published by Stewart on the insect fauna of southern Florida.

#### Comments.

In females, smaller specimens tend to have shorter wings, a smaller areolet and pterostigma in the fore wing, and lighter color than larger specimens. Males also show variation in the extent of darker areas, but are always darker than females. Although numerous specimens (60) were found and are included as part of the type series, it should be noted that all of them were collected during two collecting trips made in 1985 and 1986 (see Peck (1989) for details). Based on the specimens collected, males outnumber females in a proportion of 14: 1. This is an artifact of the collecting method used (a Malaise trap), which is better suited for rather strong fliers. Evidently females do not fly well, if at all.

## Discussion

### Brachyptery and Microgastrinae?

Belokobylskij and Kula (2012) analyzed the species of Braconidae that could be considered as apterous, micropterous and brachypterous and listed over one hundred species worldwide. Quicke (2015) commented that brachyptery occurs in a few members of virtually all braconid subfamilies within the cyclostome lineage but only in four subfamilies of non-cyclostome, none of them belonging to Microgastrinae; he also speculated that apterygy and brachyptery must have evolved many times (at least 48 times, probably more) within Braconidae.

According to Belokobylskij and Kula (2012: 6) the following parameters characterize specimens of Braconidae as brachypterous: fore wing length greater than 0.25 mesosoma length but less than metasoma length, venation distinct but incomplete, tegula present and usually not reduced in size. Females of *Keylimepie peckorum* have fore wings just slightly longer than metasoma length (1.1–1.3×), but they do not actually extend beyond T4, i.e., well before the metasoma apex (Fig. 3), and are definitely shorter (0.6–0.7×) than the body length; the venation is complete but some veins are not clearly distinct due to the reduced size of the wing, and the tegula is of normal size. They fulfill two out of three parameters proposed by Belokobylskij and Kula (2012), and thus we consider here that female specimens of *Keylimepie* are indeed brachypterous. Because wing reduction for Braconidae overall is likely continuous rather than discrete, it will be extremely difficult to strictly categorize every species as micropterous, brachypterous, and macropterous.

The phenomenon of wing size reduction in Braconidae has been related to penetration of parasitoids into the microhabitats of their hosts, temporal (seasonal) abundance of preferred hosts in limited space, or unknown causes (see Belokobylskij and Kula 2012). There may also be a tendency for short wings to evolve more on islands and in relatively harsh environments such as arid, arctic or high mountains where it is windy, hosts may be distributed in an exceedingly patchy manner, and/or due to the need to conserve energy (Quicke 2015). Specimens of *Keylimepie peckorum* have been almost exclusively collected in small islands with relatively dry environments (Deyrup et al. 1988). Two specimens were collected in mainland Florida (Everglades National Park), in a similar environment to the Florida Keys and very close to them (Fig. 18).

### Conservation of *Keylimepie peckorum* in the southern Florida environments

Because of the geographic location of the Florida Keys, its biota includes species that a) arrived naturally from nearby mainland Florida, b) arrived naturally from the nearby West Indies, c) were introduced by humans, or d) are endemic (Snyder et al. 1990, Moreau et al. 2014). Among plants, vertebrates, and butterflies, there are numerous West Indian species whose North American populations are confined just to the Florida Keys and small nearby areas of mainland Florida (Snyder et al. 1990). However, species diversity is limited by the reduced habitat types and the long dry season during winter and spring (Deyrup et al. 1988).

Conservation efforts in the Florida Keys are mainly focused on marine life, plants such as the Key tree cactus (*Pilosocereus robinii*), and charismatic fauna such as the Key deer (*Odocoileus virginianus clavium*) and the Key Largo woodrat (*Neotoma floridana smalli*). Among insects butterflies and ants have also been studied from a conservation perspective (Deyrup et al. 1988, Moreau et al. 2014, Henry et al. 2015, Jue et al. 2015).

A recent assessment on vulnerability of 300 species in Florida found a number of species from the Keys as of high priority (Reece et al. 2013). The comments made by the authors (p. 8) about conservation of some insect species are significant: “Several species, in particular invertebrates, were ranked as at high risk of extinction, but do not receive high priority for conservation due to a lack of basic life history information. Examples include the Keys scaly cricket (*Cycloptilum irregularis*), the mangrove long-horned beetle (*Heterachthes sablensis*), and the Antillean spreadwing (*Lestes spumarius*). We do not advocate abandoning these and similar species. Nevertheless, conservation actions that target species or groups of species should, whenever possible, be based on knowledge of the life history and ecology of target species... Without life-history information that would indicate potential responses to alternative management actions, we would not give high priority for conservation action to these species, aside from protecting known occurrences. On the other hand, they should receive high priority for basic research. Thus, for many species, additional research must be conducted before conservation measures beyond protecting documented populations can be successfully implemented”.

We believe that *Keylimepie peckorum* fits the above scenario perfectly. It is not only the first new genus of Microgastrinae discovered in North America in more than 30 years, but it also represents a distinctive and arguably unique lineage among microgastrine parasitoid wasps, and the species is likely to be endemic to the Florida Keys and Everglades areas. The poor ability to fly may also make *Keylimepie peckorum* less adaptable to changes in habitat distribution due to human disturbance, climate change, etc. Based on what is already known, it could easily be considered as a high priority species. However, the lack of basic biological information (host unknown) and the uncertainty of its current presence in the Keys (the only known specimens were collected 30 years ago) is a serious obstacle towards any conservation effort. This paper has only filled the first gap, giving a name to the species and summarizing what is known to date about it. More research is needed, including collecting of fresh specimens (to prove that the species is not extinct) as well as trying to find the host caterpillars of this amazing parasitoid wasp.

## Supplementary Material

XML Treatment for Keylimepie

XML Treatment for Keylimepie peckorum
